# A Current Evidence Review From the Italian Society of Pediatric Allergology and Immunology (SIAIP) on Practical Management of Allergic Rhinitis in Children and Adolescents

**DOI:** 10.1002/clt2.70218

**Published:** 2026-11-07

**Authors:** Giorgio Ciprandi, Amelia Licari, Cristiana Indolfi, Sara Manti, Michele Miraglia del Giudice, Maria Angela Tosca, Anna Maria Zicari, Gianluigi Marseglia

**Affiliations:** ^1^ Department of Medicine and Health Sciences University of Molise Campobasso Italy; ^2^ Pediatric Unit Department of Clinical, Surgical, Diagnostic, and Pediatric Sciences University of Pavia Pavia Italy; ^3^ Pediatrics Fondazione IRCCS Policlinico San Matteo Pavia Italy; ^4^ Department of Woman and Child and General and Specialized Surgery University of Campania Luigi Vanvitelli Naples Italy; ^5^ Unit of Pediatrics Department of Human Pathology in Adult and Developmental Age “Gaetano Barresi” University of Messina Messina Italy; ^6^ Allergy Center IRCCS Istituto Giannina Gaslini Genoa Italy; ^7^ Pediatric Department La Sapienza University Rome Italy

**Keywords:** adolescents, allergen immunotherapy, allergic rhinitis, children, diagnosis, pharmacotherapy, SIAIP

## Abstract

**Background:**

Allergic rhinitis (AR) is highly prevalent in children and adolescents and impairs quality of life, sleep, and school performance, while also worsening asthma control. The last EAACI position paper on pediatric AR was published in 2013. Significant scientific advancements, including the new ARIA 2026 guidelines, necessitate an updated national framework.

**Objectives:**

This Italian Society of Pediatric Allergology and Immunology (SIAIP) document aims to provide an evidence‐based age‐specific framework for the diagnosis, classification, and practical management of AR in children and adolescents, while addressing the limitations of existing less granular guidelines.

**Methods:**

A dedicated SIAIP experts' board reviewed current scientific literature, integrated updated epidemiological data, revised classifications, and emerging evidence on allergen immunotherapy. It incorporates the ARIA 2026 guidelines and adapts them to the clinical challenges and age‐specific considerations relevant to pediatric practice. The board of qualified experts and a panel of multidisciplinary clinicians discussed and approved the document.

**Results:**

This document covers the epidemiology, pathophysiology, and diagnosis of AR in children and adolescents. It provides detailed stepwise pharmacologic recommendations and discusses the role of allergen immunotherapy (AIT), taking age‐specific factors and complementary therapies into account. Evidence‐based statements and practical management algorithms aim to optimize diagnostic pathways and therapeutic interventions.

**Conclusions:**

This SIAIP document offers clinically actionable guidance for healthcare professionals. It emphasizes early diagnosis, stepwise treatment strategies, and the disease‐modifying role of AIT, aiming to enhance diagnostic accuracy, streamline management, and ultimately improve the long‐term health and well‐being of affected children and adolescents.

## Graphical abstract

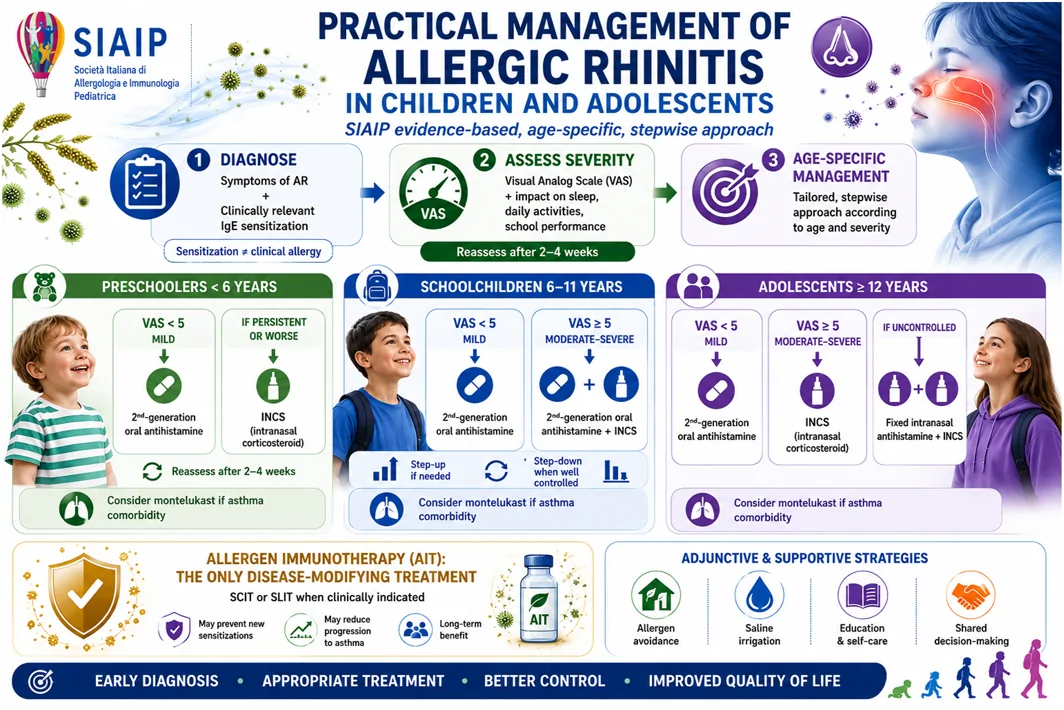

## 1 Introduction

Allergic rhinitis (AR) is a highly prevalent chronic inflammatory disease that affects an estimated 10%–40% of the pediatric population worldwide [1]. AR symptoms significantly impair quality of life, sleep, and academic performance, and can exacerbate asthma. The concept of “united airway disease” emphasizes the close link between upper‐ and lower‐airway inflammation and highlights the impact of AR on asthma symptoms in affected children [2].

The landscape of allergic disease management has evolved considerably since the last comprehensive position paper on AR in the Italian pediatric context, published in 2014 [3]. That document was based on, and commented on, the European Academy of Allergy and Clinical Immunology (EAACI) position paper on the management of pediatric AR [4]. This decade‐long gap necessitates an update, especially with the recent Allergic Rhinitis and its Impact on Asthma (ARIA) 2026 guidelines [5]. The Italian Society of Pediatric Allergology and Immunology (SIAIP) therefore developed this document to adapt international evidence‐based recommendations to the Italian healthcare setting, providing a holistic, stepwise approach to AR in children and adolescents. It covers updated epidemiology, pathophysiology, classification, diagnostic pathways, pharmacotherapy, and allergen immunotherapy (AIT), aiming to enhance diagnostic accuracy and improve long‐term health outcomes for young individuals. The process by which the evidence was selected and the recommendations formulated was based on a search of publications available on PubMed/Medline, Scopus, and Web of Science. A board of experts (i.e., the full authors) reviewed and incorporated the most important publications from an evidence‐based medicine perspective and drafted the text following in‐depth, iterative discussion.

Furthermore, a multidisciplinary panel (pediatricians, allergists, immunologists, and otorhinolaryngologists) evaluated, discussed, and approved the document. As a result, the proposed recommendations should be interpreted as evidence‐informed practical statements. Although this document is specifically tailored to the Italian healthcare context, the evidence‐based recommendations and practical algorithms presented herein are grounded in internationally recognized guidelines (including ARIA 2026 and EAACI position papers). They are therefore broadly applicable to other European and non‐European countries, with appropriate adaptation to local regulatory frameworks, drug availability, and healthcare systems.

## 2 Definition and Classification of Allergic Rhinitis

Allergic Rhinitis is an IgE‐mediated inflammatory disorder of the nasal mucosa. Pediatric practice faces challenges in symptom reporting, requiring careful assessment of pruritus, sneezing, rhinorrhea, and congestion. These nasal complaints are often accompanied by ocular manifestations (conjunctival erythema, itching, tearing, photophobia) [6].

The ARIA classification defines duration as intermittent (< 4 days/week OR < 4 consecutive weeks) or persistent (≥ 4 days/week AND ≥ 4 consecutive weeks) [7]. Severity is mild (normal sleep, no impairment of daily activities/work/school, no troublesome symptoms) or moderate‐severe (abnormal sleep, impairment of daily activities/work/school, or troublesome symptoms), based on quality‐of‐life impact [7].

Current understanding of AR increasingly incorporates phenotypes and endotypes, including type 2 inflammatory patterns, which may support future precision‐medicine approaches [8].

## 3 Epidemiology of Allergic Rhinitis in Children and Adolescents

Allergic Rhinitis (AR) represents a major global health burden in pediatric populations, with prevalence rates from 10% to 40% worldwide [1]. Large European studies like ISAAC report high AR symptoms among school‐aged children, with Italy often showing figures in the upper range [9]. Its prevalence has increased over recent decades. AR is less common in infants, rises during preschool, peaks in school‐age children and adolescents, and shows a slight male predominance in childhood. Its development reflects the interaction between genetic predisposition, including a strong family history of atopy, and environmental factors such as air pollution, tobacco smoke, and climate change (Figure 1). The “hygiene hypothesis” and disturbances in the microbiome also contribute to changes in prevalence [10]. In Italy, distinguishing seasonal (pollen) from perennial (dust mites, pet dander) AR is crucial for effective management of children [11].

**FIGURE 1:**
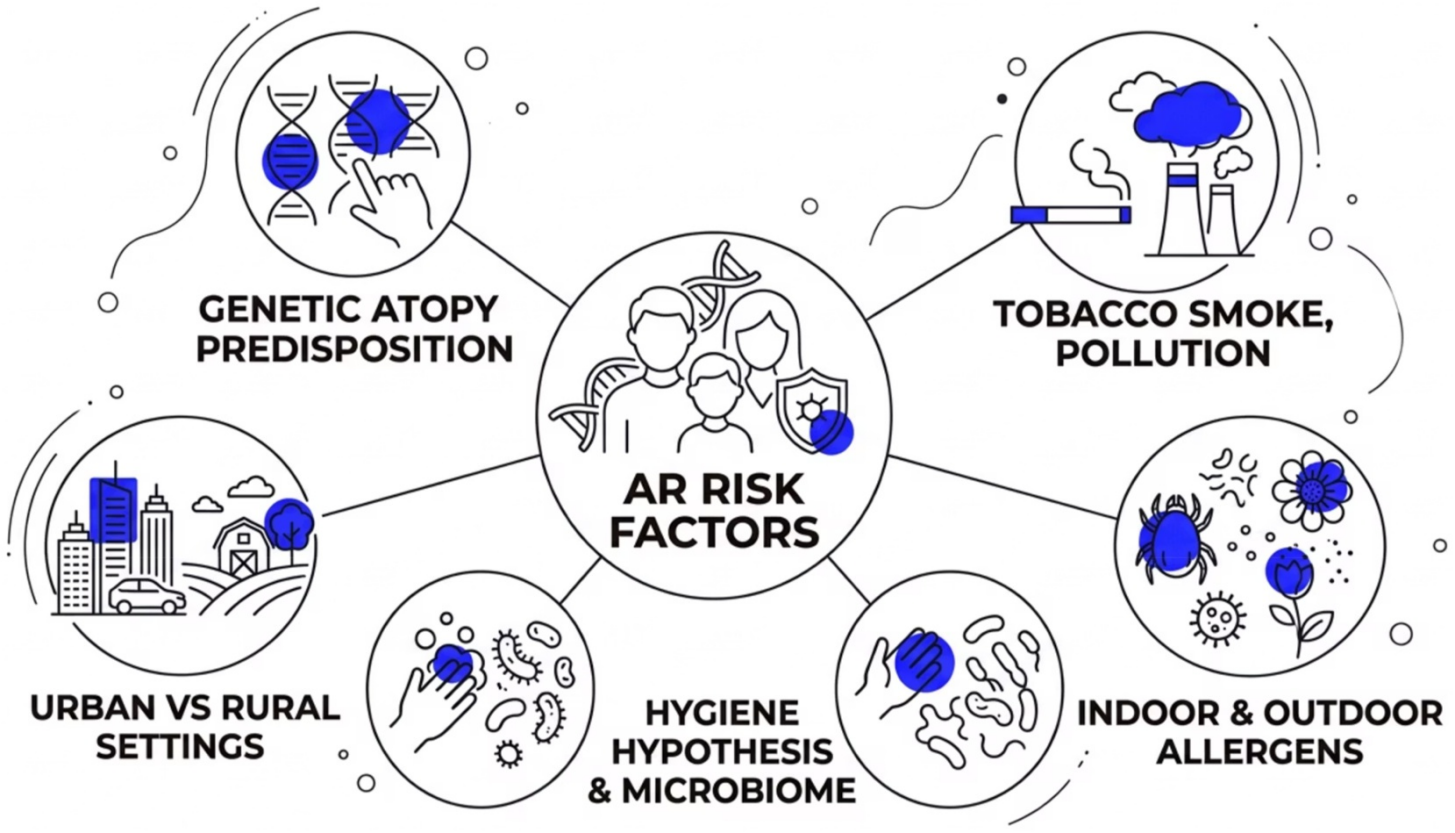
Etiology of AR: Multifactorial, involving a complex interplay of genetic and environmental influences.

## 4 Socioeconomic Burden of Allergic Rhinitis in the Pediatric Population

AR in children and adolescents imposes a substantial socioeconomic burden, as summarized in Figure 2 [12]. Direct costs include medical consultations, medications, diagnostic tests, and emergency visits. Indirect costs arise from parental work loss and school absenteeism, with consequent effects on educational performance and family functioning. Persistent symptoms such as nasal congestion and sleep disturbances lead to fatigue, impaired concentration, and memory difficulties, negatively affecting school performance and long‐term educational trajectories. AR also burdens families with children's sleep disturbances, social embarrassment, and parental stress [13]. European data estimate the annual per‐patient cost to be €500‐ €1500 [14]. However, early intervention and allergen immunotherapy are cost‐effective strategies that offer long‐term benefits, including improved symptom control and reduced healthcare utilization.

**FIGURE 2:**
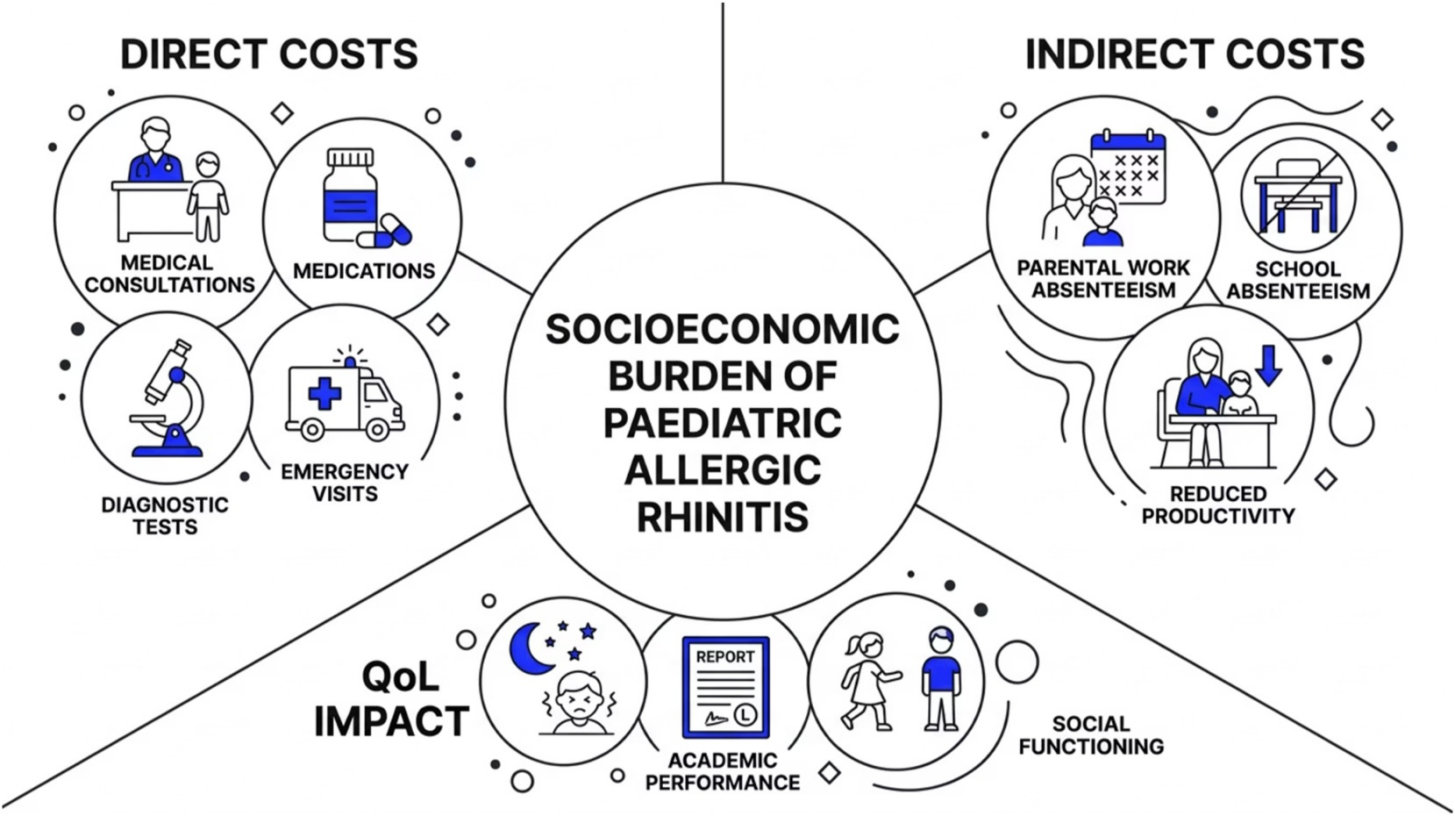
Socioeconomic burden of pediatric AR.

## 5 Pathophysiology of Allergic Rhinitis in Children

AR in children is a complex IgE‐mediated inflammatory disease of the nasal mucosa [15]. Table 1 summarizes the most important pro‐inflammatory mediators involved in AR pathogenesis. The sensitization phase begins with allergen exposure, during which antigen‐presenting cells (APCs) drive T helper 2 (Th2) and innate lymphocyte cell (ILC2) differentiation [16]. These Th2 and ILC2 cells secrete cytokines (IL‐4, IL‐5, IL‐13), which promote B cell activation and the production of allergen‐specific immunoglobulin E (IgE) antibodies [17]. IgE then primes mast cells in the nasal mucosa for future allergen encounters. The effector phase initiates upon re‐exposure to the allergen. Allergen binding to IgE on mast cells triggers rapid degranulation and release of inflammatory mediators, marking the immediate‐phase reaction. Table 1 summarizes key pro‐inflammatory mediators involved in the pathophysiology of allergic rhinitis and their primary effects. Histamine causes vasodilation, increased vascular permeability, glandular secretion (rhinorrhea), pruritus, and sneezing. Leukotrienes contribute to increased vascular permeability and mucus secretion, while prostaglandins induce vasodilation and nerve stimulation, collectively causing classic AR symptoms. A prolonged late‐phase reaction (4–12 hours) is characterized by marked cellular infiltration, notably eosinophils, leading to tissue damage, inflammation, and nasal hyperresponsiveness. This process also causes epithelial barrier dysfunction and impairs mucociliary clearance.

**TABLE 1:** Main pro‐inflammatory mediators and primary effects in allergic reaction.

Mediator	Primary effects
Histamine	Vasodilation, increased vascular permeability, glandular secretion, pruritus, sneezing, smooth muscle contraction
Prostaglandin D2 (PGD2)	Increased vascular permeability, mucus secretion, bronchoconstriction
Leucotrienes (LTC4, LTD4, LTE4)	Vasodilation, plasma exudation, nerve stimulation, mast cell activation
Tryptase	Tissue remodeling, mucus secretion, airway hyperresponsiveness
Type 2 cytokines (IL‐4, IL‐5, IL‐13)	IgE production, eosinophil recruitment and activation, mucus production, Th2 differentiation
Chemokines (e.g., eotaxin)	Recruitment of eosinophils and other inflammatory cells
Major basic protein (MBP)	Epithelial damage, neurotoxicity
Eosinophil cationic protein (ECP)	Epithelial damage, enzyme release
Platelet activating factor PAF	Vascular permeability, nasal congestion, rhinorrhea, bronchoconstriction, bronchial hyperresponsibveness, mucus secretion, histamine release

## 6 Complications and Comorbidities of Allergic Rhinitis in Children

Children with AR may show different clinical trajectories and patterns of comorbidity [18]. AR is frequently associated with significant complications and comorbidities, as summarized in Figure 3 [19]. Chronic inflammation contributes to chronic rhinosinusitis by obstructing sinus ostia. Eustachian tube dysfunction causes otitis media with effusion (OME) and conductive hearing loss [20]. Nasal obstruction leads to sleep‐disordered breathing, mouth breathing, fragmented sleep, fatigue, attention deficits, and academic impact [21]. The “united airway” concept highlights the strong association between AR and asthma, with AR contributing to poorer control of lower airway inflammation [5]. Persistent mouth breathing from AR can alter craniofacial growth patterns, contributing to “adenoid facies.” AR also frequently coexists with allergic conjunctivitis. Furthermore, AR significantly impacts psychological and neurocognitive well‐being, causing ADHD‐like symptoms, anxiety, and depression [22].

**FIGURE 3:**
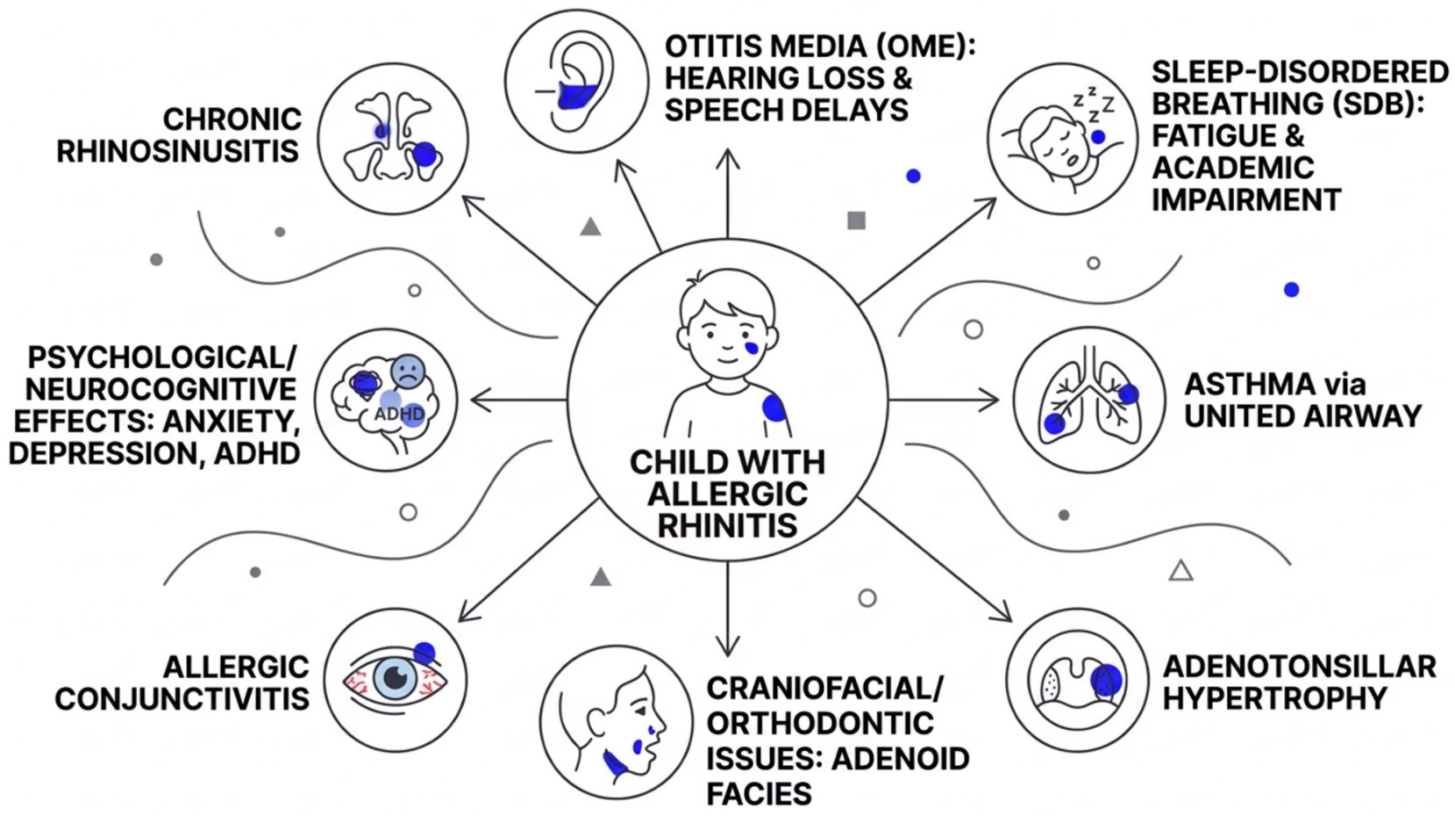
Main comorbidities in children and adolescents with AR.

## 7 Diagnosis of Allergic Rhinitis in Children

Accurate diagnosis of AR in children is crucial for effective management and the prevention of complications, as underscored by a recent Delphi Consensus [23]. A comprehensive approach integrates clinical history, physical examination, and objective diagnostic tests, tailored to pediatric patients. Systematic evaluation is essential to differentiate AR from other forms of rhinitis, given varied presentations and potential overlaps [24].

A careful clinical history should include assessment of symptoms such as rhinorrhea, nasal congestion, sneezing, and pruritus. Inquiring about symptom timing helps differentiate seasonal (pollen) from perennial (dust mites, pet dander) patterns. Family history of atopy and the impact on sleep, school, and daily activities further guide diagnosis and assess severity.

Physical examination: The nasal passages and surrounding structures should be carefully examined. Typical findings include pale, boggy nasal turbinates with clear discharge. Suggestive signs such as “allergic shiners” and a “nasal crease” may be present, along with mouth breathing. Examination also assesses for Eustachian tube dysfunction or otitis media with effusion, which are common comorbidities.

The differential diagnosis of rhinitis in children is broad, including non‐allergic rhinitis, infectious rhinitis, and structural abnormalities. Non‐allergic rhinitis mimics AR symptoms but lacks an allergic etiology. Infectious rhinitis presents with acute onset, fever, and purulent discharge, while structural issues like adenoid hypertrophy cause chronic obstruction and must be ruled out.

Allergy Testing: Skin prick testing (SPT) and specific IgE serum testing are both well‐established and equally valid first‐line methods for documenting IgE‐mediated sensitization, each offering distinct advantages and limitations. SPT involves applying an allergen to the skin and observing wheal‐and‐flare reactions; it is reliable from infancy but requires discontinuation of antihistamines [25]. Specific IgE serum testing measures circulating IgE antibodies via a blood test and is independent of skin reactivity or medication use, making it particularly useful when SPT is not feasible [26]. Clinical context, patient characteristics, and local availability should guide the choice between the two methods.

Component‐Resolved Diagnostics (CRD) offers advanced molecular allergy diagnosis by measuring specific IgE antibodies to individual allergenic components [27]. This approach allows more precise identification of primary sensitizers and helps distinguish true sensitization from cross‐reactivity. In polysensitized children, CRD is valuable for prioritizing relevant allergens for avoidance and for selecting immunotherapy.

A key concept should be emphasized: skin tests and serum‐specific IgE assays document sensitization, not clinical allergy. However, sensitization does not necessarily imply clinically relevant allergy. Clinical allergy is established only when exposure to the relevant allergen consistently triggers symptoms. It is a common mistake to confuse sensitization with allergy. Therefore, the diagnostic process must ultimately establish whether sensitization is clinically relevant.

### 7.1 Ancillary Diagnostic Tools

Nasal cytology involves microscopic examination of nasal secretions to identify inflammatory cells [28]. The presence of eosinophils strongly suggests AR or non‐allergic eosinophilic rhinitis. Although simple to perform, its stand‐alone diagnostic value is limited, serving primarily as an adjunct.

Nasal Provocation Testing (NPT) involves applying allergens directly to the nasal mucosa to confirm clinical reactivity, though it is rarely used in routine pediatric practice. NPT may be useful for identifying local allergic rhinitis [29]. It is also reserved for uncertain diagnoses or for assessing the relevance of immunotherapy, given its time‐consuming nature and the need for specialized settings.

Quality‐of‐life tools: Standardized Quality‐of‐Life (QoL) instruments, such as the Pediatric Rhinoconjunctivitis Quality of Life Questionnaire (PRQLQ), quantify the burden of AR on physical, emotional, social, and academic functioning [30]. These questionnaires provide valuable subjective insights from children and parents, complementing objective findings and guiding monitoring of treatment effectiveness.

## 8 Treatment of Allergic Rhinitis in Children

The pharmacological management of allergic rhinitis in children requires a systematic, stepwise approach tailored to the child's age, symptom severity, and overall clinical profile. Current guidelines, such as those from ARIA 2026, advocate a treatment strategy that begins with the least invasive yet effective options and escalates as needed to ensure optimal symptom control while minimizing potential side effects [5, 30, 31, 32, 33, 34, 35]. This approach prioritizes patient safety and efficacy, recognizing the long‐term implications of AR on a child's development and quality of life. However, these relevant documents do not adequately address pediatric aspects of AR, as highlighted by recent reviews [36, 37, 38]. Notably, the EAACI position paper remains the most specific reference document for pediatric AR management [4]. For this reason, the SIAIP has promoted a series of initiatives to provide practical recommendations on the treatment of AR in pediatrics. In particular, a Delphi Consensus and a Survey revealed the need to develop practical recommendations for therapeutic management [11, 23]. A recent SIAIP position paper also highlighted the clinical relevance of fixed intranasal combination therapy [39]. Furthermore, it is also important to consider two fundamental aspects: (i) the therapeutic management of children and adolescents cannot be transferred outright from the guidelines targeted for adult patients, and (ii) each country has its own specific characteristics (e.g., diet, lifestyle, public health, economic sustainability, available medications, and cultural level), which influence therapeutic choices and therefore require local adaptation of the guidelines. As a result, the present document aims to provide practical recommendations for the treatment of AR in children and adolescents in the Italian context.

From a clinical point of view, an appropriate AR treatment should be based on a precise diagnosis, an assessment of symptom severity, an assessment of comorbidities, and the prescription of a tailored therapy that may include prevention measures, drugs, complementary remedies, allergen‐specific immunotherapy (AIT) if indicated, and educational issues. AR management should also be based on shared decision‐making, including patient and family engagement, education activities, a complete explanation of measures, an action plan, and consent. This approach may improve adherence and reduce the risk of adverse events. This approach reflects a patient‐centered model of care [40, 41]. In addition, the choice of the best treatment should take into account the pathophysiologic mechanisms underlying each AR patient's condition. A practical distinction between predominantly histamine‐dependent symptoms (itching, sneezing, rhinorrhea, and congestion) and predominantly inflammation‐dependent symptoms (obstruction, olfactory dysfunction, and sleep disorders) may help guide pharmacologic choice. Phenotypic assessment may also be helpful, including symptom duration and susceptibility to respiratory infections. A close link between allergy and infection exists, as documented by the concept of minimal persistent inflammation and relative immune defect in allergic subjects [42]. Allergic reaction is characterized by type 2 inflammation, which is closely dependent on allergen exposure [43]. In other words, exposure to the causal allergen causes and maintains inflammation. Inflammatory events even include the expression of adhesion molecules (mainly ICAM‐1) that, on one hand, mediate the inflammatory infiltrate and, on the other hand, may promote viral infections (ICAM‐1 is the main receptor of rhinoviruses) [44]. In addition, type 2 inflammation depends on T helper 2 polarization, which, in turn, leads to defective type 1 immune responses, which are involved in host defense against infection [45]. As a result, children (mainly) and adolescents with allergies are prone to frequent infections and chronic allergies [46]. Therefore, appropriate treatment of pediatric AR requires management based on these grounds. Traditionally, AR therapy includes prevention, pharmacotherapy, AIT, and ancillary remedies.

### 8.1 Preventive Measures

Avoidance of clinically relevant allergens would ideally reduce symptom burden and inflammatory activation in AR [47]. Therefore, an allergic individual who completely avoids exposure will have no symptoms. However, complet. allergen avoidance is rarely achievable in real life. Certainly, the opposite reasoning applies: the greater the allergen exposure and the longer the allergic person is exposed to it, the more severe the AR will be. Therefore, it is advisable to avoid exposure to high concentrations of the causative allergen whenever possible. Pollen count calendars and forecasts provide one example. If a high pollen concentration is expected, especially depending on the weather, it is best to avoid being outdoors or to wear a mask. As for household allergens, it is important to reduce as much as possible the environmental conditions that favor excessive mite and mold growth (humidity and temperature). For pet allergens, avoidance measures are often difficult to implement, particularly because of the emotional bond between the child and the animal. In general, preventive measures can yield results, but they cannot eliminate allergies. It is for this reason that drug therapies are often necessary.

### 8.2 Pharmacologic Treatment of Allergic Rhinitis in Children

Pharmacologic treatment of pediatric AR includes several drug classes, although antihistamines and intranasal corticosteroids (INCS) remain the most effective and safest options. In particular, antihistamines are indicated for histamine‐dependent symptoms, whereas corticosteroids are indicated for inflammation‐mediated complaints [32]. The other classes are less reliable either because of their lower efficacy, higher rates of adverse events, or lack of regulatory therapeutic indication. The main pharmacological classes are reported in Figure 4.

– Second‐generation oral antihistamines (e.g., bilastine, cetirizine, desloratadine, ebastine, levocetirizine, loratadine, fexofenadine, and rupatadine) are cornerstones of AR treatment, primarily acting through H1 blockade [48]. They effectively alleviate sneezing, itching, and rhinorrhea, but are less effective for nasal congestion. Preferred over first‐generation agents due to significantly reduced sedative and anticholinergic effects, their favorable sedative profile makes them less likely to interfere with daily activities and school performance. Age‐specific dosing and formulations, such as liquid preparations, are critical and based on the child's weight and developmental stage. Table 2 reports the principal characteristics of oral antihistamines.
– Intranasal antihistamines include azelastine and olopatadine; however, only azelastine nasal spray is currently available in this setting. It offers a rapid onset of action (15–30 minutes) and provides quick relief of sneezing, itching, and rhinorrhea [49]. It has a rapid onset of action and may be used on demand. In current practice, it is approved for use in children aged 6 years and older [39].
– INCS are the first‐line therapy for children with moderate‐to‐severe or persistent AR and are widely regarded as the most effective agents for controlling the full spectrum of AR symptoms, particularly nasal obstruction, which reflects underlying inflammation [50]. Their mechanism involves potent anti‐inflammatory effects within the nasal mucosa. Modern INCS formulations have minimal systemic bioavailability, making them generally safe for long‐term use in children [51, 52]. Concerns regarding their impact on growth velocity have been largely allayed by studies showing negligible clinical effects at recommended doses, particularly for newer‐generation INCS [53]. Proper administration technique is crucial: tilt the head forward, aim away from the nasal septum, and avoid forceful sniffing after actuation [54]. Table 3 reports the principal characteristics of intranasal corticosteroids.
– A fixed combination of intranasal antihistamine with intranasal corticosteroid is an effective option to control moderate‐severe symptoms in AR adolescents, as recently outlined in some documents [39, 55, 56]. However, the current formulations are not indicated for use in children, despite evidence of their effectiveness, and regulatory approval is still pending in the European Union. Presently, two combinations are available: azelastine/fluticasone and olopatadine/mometasone.
– Leukotriene receptor antagonists (LTRAs), primarily montelukast, target inflammatory mediators involved in allergic reactions. Montelukast is approved for seasonal AR treatment in children as young as 6 months and is particularly beneficial for patients with concomitant asthma, addressing both conditions concurrently [57]. While generally well‐tolerated, rare neuropsychiatric events have been reported, necessitating careful consideration of its use.
– Decongestants provide rapid relief from nasal congestion but have a limited role in pediatric AR due to potential side effects. Oral decongestants carry risks of central nervous system stimulation and are not recommended for children. Topical decongestants should be used for no more than 3–5 days to avoid rhinitis medicamentosa, a rebound congestion phenomenon [4, 36]. Their use is strictly managed and reserved for acute, severe congestion.
– Chromones, like sodium cromoglycate and nedocromil, are mast cell stabilizers with a good safety profile but limited efficacy compared to INCS and antihistamines, requiring frequent dosing [58]. Consequently, their use has largely declined in pediatric AR management, primarily reserved for very mild, intermittent symptoms or as an alternative for patients who cannot tolerate other treatments.
– Anticholinergics, such as intranasal ipratropium bromide, selectively block cholinergic receptors, effectively reducing watery rhinorrhea [59]. They are primarily indicated for patients whose predominant symptom is severe watery rhinorrhea, with minimal efficacy for other AR symptoms. However, there is no indication for the pediatric population.
– Biologics are a continuously increasing class of monoclonal antibodies targeting different pathways in type 2 inflammation. However, there is no indication for AR. Anyway, children with AR and coexisting severe allergic asthma or atopic dermatitis significantly benefited from biologics [60].

**FIGURE 4:**
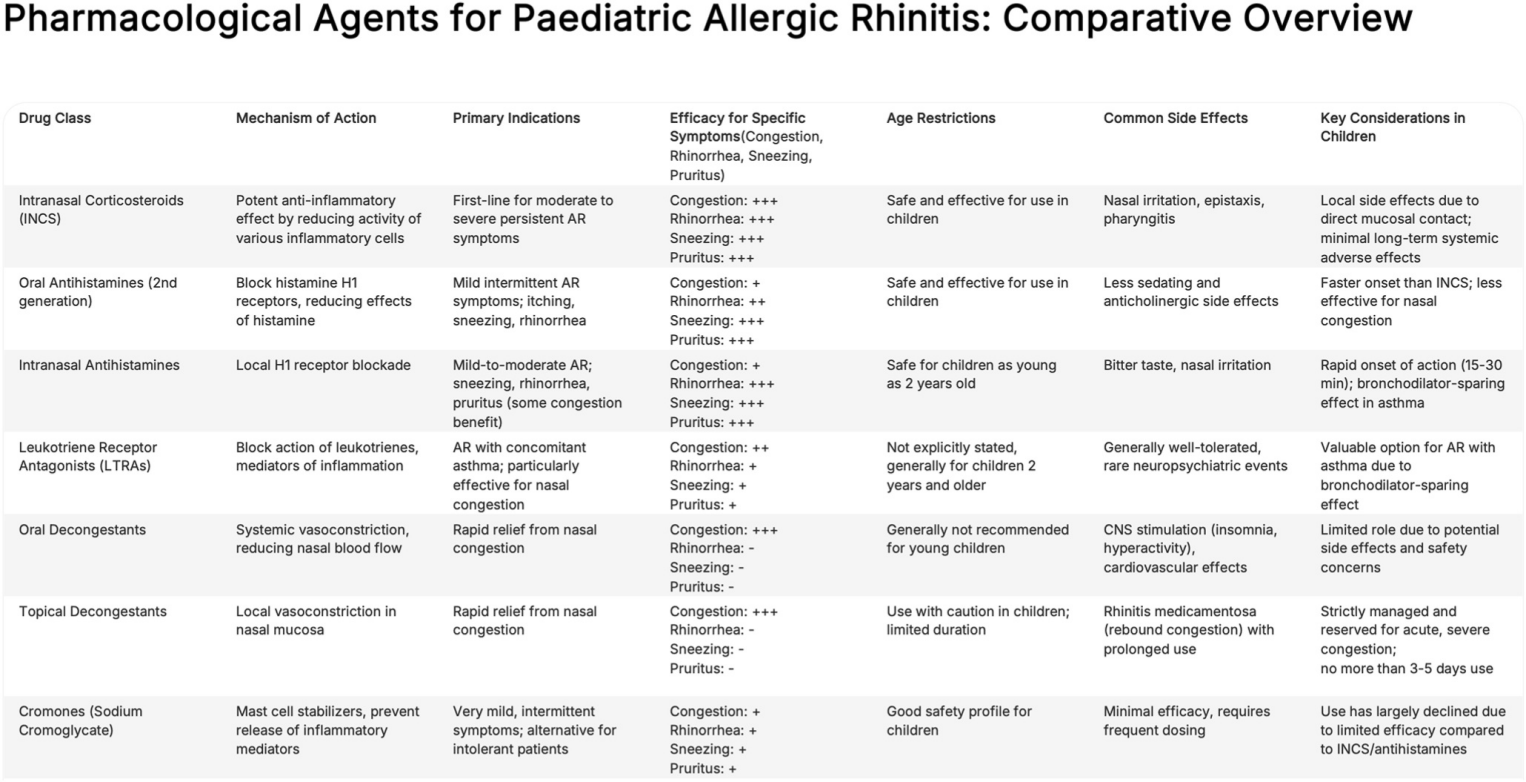
Medications for treating AR in children and adolescents.

**TABLE 2:** Oral second ‐generation antihistamines used in AR treatment.

Molecule	Formulation	Posology	Age	Indication
Bilastine	Fast‐dissolving tablet 10 mg	10 mg/die	6–11 years (> 20 kg/bw)	SAR/PAR
	Tablet 20 mg	20 mg/die	Adolescents	SAR/PAR
Cetirizine	Tablet 10 mg	10 mg/die	Ages 6 and up	AR
	Drop 10 mg/mL	2.5 mg/die (5 drops)	2–6 years	AR
Desloratadine	Oral solution 0.5 mg/mL	1.25 mg/die 1–5 years 2.5 mg/die 6–12 years	1–12 years	IAR ‐per
	Fast‐dissolving tablet		Adolescents	IAR‐PER
	Tablet 5 mg	5 mg/die	Adolescents	IAR‐PER
Ebastine	Tablet 10 mg Fast‐dissolving tablet 10 mg	10–20 mg/die	Adolescents	SAR/PAR
Fexofenadine	Tablet 120 mg	120 mg/die	Adolescents	AR
Levocetirizine	Drop 5 mg/mL	2.5 mg/die 2–6 years 5 mg/die 6–12 years	2–12 years	SAR/PAR
	Tablet 5 mg	5 mg/die	Adolescents	SAR/PAR
Loratadine	Syrup 1 mg/mL	< 30 kg/bw 5 mL > 30 kg/bw 10 mL	2–12 years	AR
	Tablet 10 mg	10 mg/die	Adolescents	AR
Rupatadine	Oral solution 1 mg/mL	10–25 kg/bw 2.5 mL ≥ 25 kg/bw 5 mL	2–11 years	
	Tablet 10 mg	10 mg/die	Adolescents	AR

Abbreviations: bw = body weight; IAR = intermittent allergic rhinitis; PAR = perennial allergic rhinitis; PER = persistent allergic rhinitis; SAR = seasonal allergic rhinitis.

**TABLE 3:** Intranasal corticosteroids prescribed in treating children and adolescents with AR.

Molecule	Formulation	Posology	Age	Indication
Beclomethasone	100 mcg/spray	1‐2 spray/nostril once a day	≥ 6 years	SAR/PAR
Budesonide	50 mcg/spray	1‐2 spray/nostril twice a day	> 6 years	SAR/PAR
Flunisolide	25 mcg/spray	1 spray/nostril three times a day	> 5 years	SAR/PAR
Fluticasone furoate	27.5 mcg/spray	1 spray/nostril once a day 2 spray/nostril once a day	6–11 years adolescents	SAR/PAR
Fluticasone propionate	50 mcg/spray	1 spray/nostril once a day 2 spray/nostril once a day	4–17 years adolescents	SAR/PAR
Mometasone	50 mcg/spray	1 spray/nostril once a day 2 spray/nostril once a day	3–11 years adolescents	SAR/PAR
Triamcinolone	55 mcg/spray	1 spray/nostril once a day 1‐2 spray/nostril once a say 2 spray/nostril once a day	2–5 years 6–12 years adolescents	SAR/PAR

Combination therapy strategies are frequently employed when monotherapy fails [31, 32]. The most common and effective approach is to combine INCS with an oral or intranasal antihistamine, leveraging their distinct mechanisms to provide comprehensive relief. Another valuable combination is INCS plus an LTRA, especially in children with comorbid asthma.

Throughout all interventions, meticulous attention to age‐specific dosing and formulations is paramount. Children's pharmacokinetic and pharmacodynamic profiles differ from those of adults, requiring careful dose adjustments [37, 51]. Pediatric‐friendly formulations improve acceptance and compliance. Despite effective medications, treatment adherence challenges are common due to the chronic nature of the condition, perceived lack of efficacy, and parental concerns. Educating parents and children, simplifying regimens, and open communication are crucial for improving long‐term outcomes.

## 9 Allergen Immunotherapy in Pediatric Allergic Rhinitis

Allergen immunotherapy (AIT) is a disease‐modifying treatment for pediatric AR that induces immune tolerance toward the causal allergen. It usually involves administering gradually increasing doses of specific allergens, shifting the immune response from a pathogenic T helper 2 (Th2) phenotype to a tolerogenic T helper 1 (Th1) and T regulatory (Treg) cell response [61]. Key mechanisms include the production of blocking IgG4 antibodies, which compete with IgE for mast cell binding and reduce histamine release, and the generation of T regulatory cells that secrete immunosuppressive cytokines such as IL‐10 and TGF‐β [62].

AIT offers significant disease‐modifying effects, including long‐term symptom relief that can persist for several years after treatment cessation. It prevents the development of new sensitizations and, critically in children, can prevent the progression of AR to allergic asthma when initiated early.

Historically, subcutaneous immunotherapy (SCIT) involves administering allergen extracts via subcutaneous injections. It typically comprises a build‐up phase followed by a maintenance phase over 3–5 years. SCIT is highly effective but carries a risk of systemic reactions, including anaphylaxis, requiring administration in a medical setting with 30‐min post‐injection monitoring.

Sublingual immunotherapy (SLIT) involves placing allergen extracts (tablets or drops) under the tongue. It offers greater convenience and a more favorable safety profile, making it suitable for home administration after initial training [63]. SLIT shows comparable efficacy to SCIT, particularly for moderate‐to‐severe AR, with a significantly lower risk of systemic adverse reactions [64]. Local oral reactions (pruritus, irritation) are common initially but are usually mild and transient.

The choice between SCIT and SLIT should be individualized, taking into account factors such as the child's age, specific allergen sensitivities, symptom severity, patient/parental preferences, and access to healthcare facilities.

AIT indications in pediatric AR include moderate‐to‐severe symptoms inadequately controlled by conventional pharmacotherapy, confirmed IgE‐mediated sensitivity, and patient/family motivation for a 3–5‐year commitment. Early intervention may also prevent the onset of asthma and new sensitizations [65]. Contraindications include uncontrolled asthma, bronchial obstruction (i.e., FEV_1_ < 70%), immunodeficiency, active autoimmune diseases, malignancy, β‐blocker therapy, and pregnancy (for initiation) [66]. Allergen selection is crucial, using skin prick tests/specific IgE assays, with component‐resolved diagnostics (CRD) for polysensitised patients to ensure clinical relevance. The typical duration of AIT is 3–5 years to achieve sustained immunological tolerance and long‐term benefits.

## 10 Complementary and Adjunctive Therapies in Pediatric Allergic Rhinitis

Complementary therapies for pediatric AR aim to mitigate symptom burden. These include nasal lavage, botanicals, trace elements, vitamins, and probiotics [67]. Nasal saline irrigation (NSI) uses isotonic/hypertonic saline to remove allergens, improve mucociliary clearance, and reduce reliance on pharmacotherapy [68]. Probiotics show emerging evidence of reducing AR symptoms, but current research is insufficient to support routine recommendation [69]. Vitamin D deficiency is associated with AR risk, but the evidence is not conclusive for routine supplementation [70]. Trace elements, particularly zinc, may be a valuable option, as may lactoferrin [71]. Botanical remedies have limited evidence, despite several real‐world studies reporting interesting outcomes [72]. In this regard, a multicomponent food supplement containing quercetin, perilla, and vitamin D has evidence supporting its efficacy and safety as an add‐on therapy in children with AR, as documented by RCTs [67, 73, 74, 75, 76, 77, 78].

## 11 SIAIP Recommendations for the Practical Management of AR

This document provides a decalogue of evidence‐based statements for the management of AR in children and adolescents, summarizing key clinical recommendations supported by current research.

1. *Allergic rhinitis in children should be classified according to the ARIA 2026 criteria based on duration (intermittent* vs*. persistent) and severity (mild* vs*. moderate‐severe), with severity assessed by impact on sleep, daily activities, and school performance rather than symptom intensity alone. A visual analog scale (VAS) is a validated tool for measuring symptom severity and for orienting treatment.* Rationale: The ARIA 2026 guidelines provide a clinically relevant framework for guiding treatment decisions and better reflecting disease burden in AR patients.
2. *Skin prick testing (SPT) and specific IgE serum testing are both well‐established and equally valid first‐line methods for confirming IgE‐mediated sensitization in children with suspected AR, each offering distinct advantages and limitations. SPT involves applying an allergen to the skin and observing wheal‐and‐flare reactions; it is reliable from infancy but requires discontinuation of antihistamines. Specific IgE serum testing measures circulating IgE antibodies* via *a blood test and is independent of skin reactivity or medication use. Clinical context, patient characteristics, and local availability should guide the choice between the two methods.* Rationale: Both SPT and serum‐specific IgE testing are complementary and equally valid diagnostic tools; neither should be ranked over the other, as each has unique advantages and limitations depending on the clinical setting.
3. *Component‐resolved diagnostics (CRD) should be considered in polysensitized children to identify primary sensitizers, distinguish true sensitization from cross‐reactivity, and guide allergen selection for immunotherapy.* Rationale: CRD provides precision in allergen characterization that whole‐extract testing cannot achieve, particularly relevant for AIT candidate selection.
4. *Allergic rhinitis diagnosis is based on demonstrating the clinical relevance of sensitization, such as documentation that exposure to the sensitizing allergen causes symptoms.* Rationale: allergen exposure causes an inflammatory reaction that sustains symptom occurrence,
5. *Second‐generation oral antihistamines are recommended for mild intermittent AR and as add‐on therapy to INCS for moderate‐severe AR, particularly when ocular symptoms are prominent; first‐generation sedating antihistamines should be avoided in children due to their adverse cognitive and sedative effects.* Rationale: Non‐sedating antihistamines have a well‐established safety profile and do not impair school performance or daily functioning.
6. *Intranasal corticosteroids (INCS) are the most effective pharmacological treatment for moderate‐severe and persistent AR in children and should be considered first‐line therapy; modern formulations (mometasone furoate, fluticasone propionate) are safe for long‐term use with no clinically significant impact on growth at recommended doses.* Rationale: Multiple RCTs and meta‐analyses confirm INCS superiority over antihistamines for overall symptom control, including nasal congestion.
7. *Leukotriene receptor antagonists (montelukast) represent a potential therapeutic option in children with concomitant AR and asthma, providing dual symptomatic relief for both conditions; neuropsychiatric adverse events should be discussed with families before initiation.* Rationale: Montelukast is approved for use in children aged 6 months and older and addresses both upper‐ and lower‐airway inflammation through the unified airway concept.
8. *In adolescents with moderate‐severe AR inadequately controlled by monotherapy, a combination of INCS with an intranasal antihistamine is recommended and has demonstrated superior efficacy compared to either agent alone. The combinations azelastine/fluticasone and olopatadine/mometasone are effective and safe.* Rationale: Combination therapy targets multiple inflammatory pathways simultaneously, providing more comprehensive symptom relief.
9. *Allergen immunotherapy (AIT), both subcutaneous (SCIT) and sublingual (SLIT), is the only disease‐modifying treatment for AR and should be considered in children with moderate‐to‐severe persistent AR inadequately controlled by pharmacotherapy; AIT may prevent new sensitizations and reduce the risk of progression to asthma.* Rationale: Multiple RCTs and long‐term follow‐up studies confirm AIT's disease‐modifying effects, including sustained post‐treatment benefit and asthma prevention.
10. *Allergen‐avoidance measures and nasal saline irrigation should be recommended as adjunctive strategies for all children with AR; saline irrigation reduces allergen load, improves mucociliary clearance, and decreases reliance on pharmacotherapy. Complementary treatments (including probiotics, botanicals, vitamins, oligo‐elements, and lactoferrin) may be useful as an add‐on strategy. However, only compounds with documented evidence should be used.* Rationale: Environmental control and nasal saline irrigation are safe, cost‐effective interventions that complement pharmacological management and improve overall symptom control. Several RCTs and real‐world studies have documented the efficacy and safety of certain products.

## 12 SIAIP Algorithm for the Practical Management of AR

The management of AR should consider the subject's age because clinical characteristics differ and drug availability varies. Figure 5 summarizes the different steps in managing children and adolescents with AR. In addition, three scenarios must be considered: one for preschoolers, one for schoolchildren, and one for adolescents.

**FIGURE 5:**
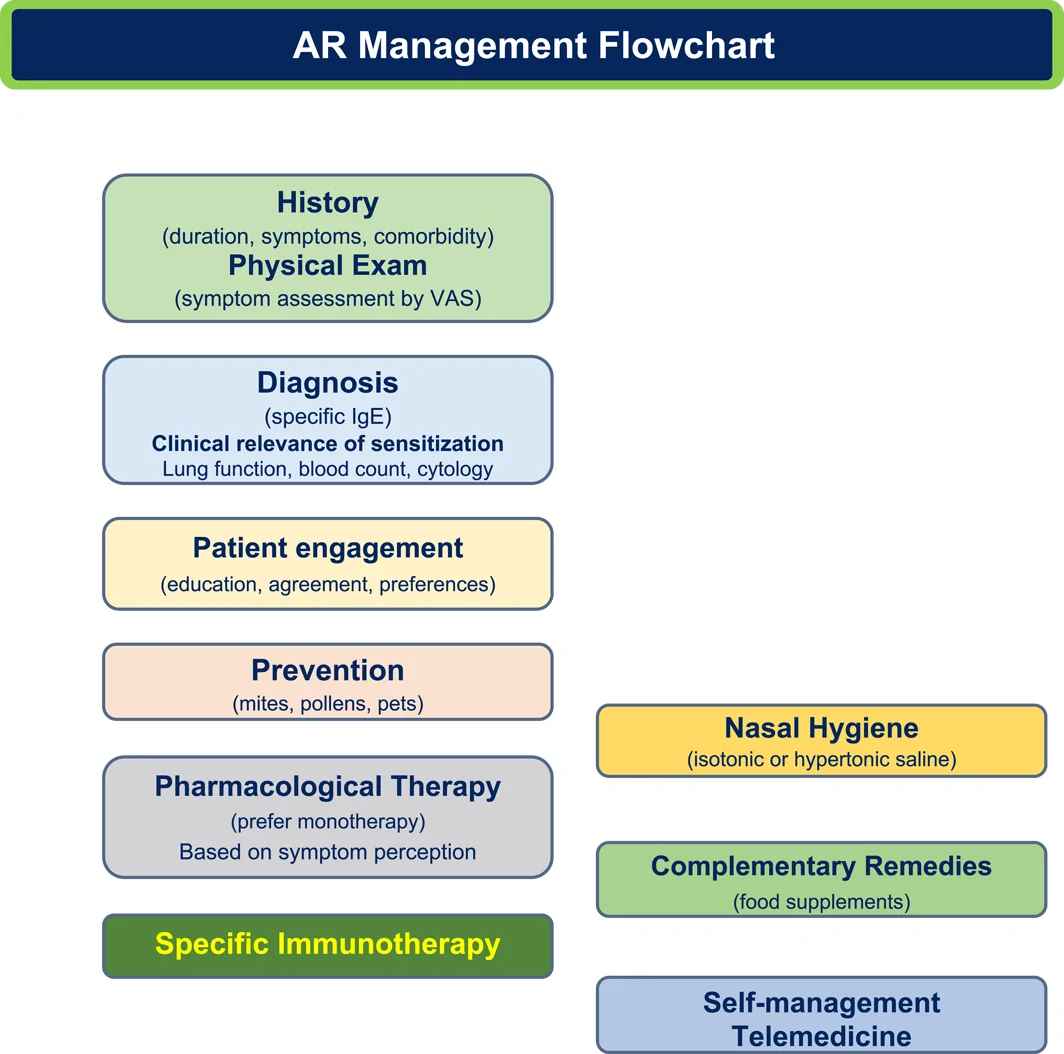
Flowchart concerning the different steps of AR management in children and adolescents.

### 12.1 Preschoolers

As summarized in Figure 6, a practical algorithm presents a flowchart useful for managing preschoolers (< 6 years) with AR. As a first step, it is fundamental to assess symptom severity to decide the best therapy. In children younger than 5–6 years, symptom severity may be assessed using a visual analog scale administered to their parents (or caregivers). Although this is an indirect method of assessing symptom intensity, it can still provide insight into not only the severity of nasal symptoms but also the impact of rhinitis on sleep, quality of life, and recreational activities. If symptoms are mild (VAS score < 5), the first‐line treatment is an oral antihistamine. However, the choice of antihistamine should consider the age indication, as some oral H1 antihistamines are indicated only in older children (see Table 2). After the initial reassessment (2–4 weeks), if symptoms persist or worsen, an intranasal corticosteroid may substitute for an oral antihistamine. The VAS score could guide treatment strategy according to a step‐up/step‐down principle. Non‐pharmacological remedies may be useful in controlling symptoms and sparing medications. However, the choice should always be based on documented evidence. Asthma comorbidity may warrant additional montelukast.

**FIGURE 6:**
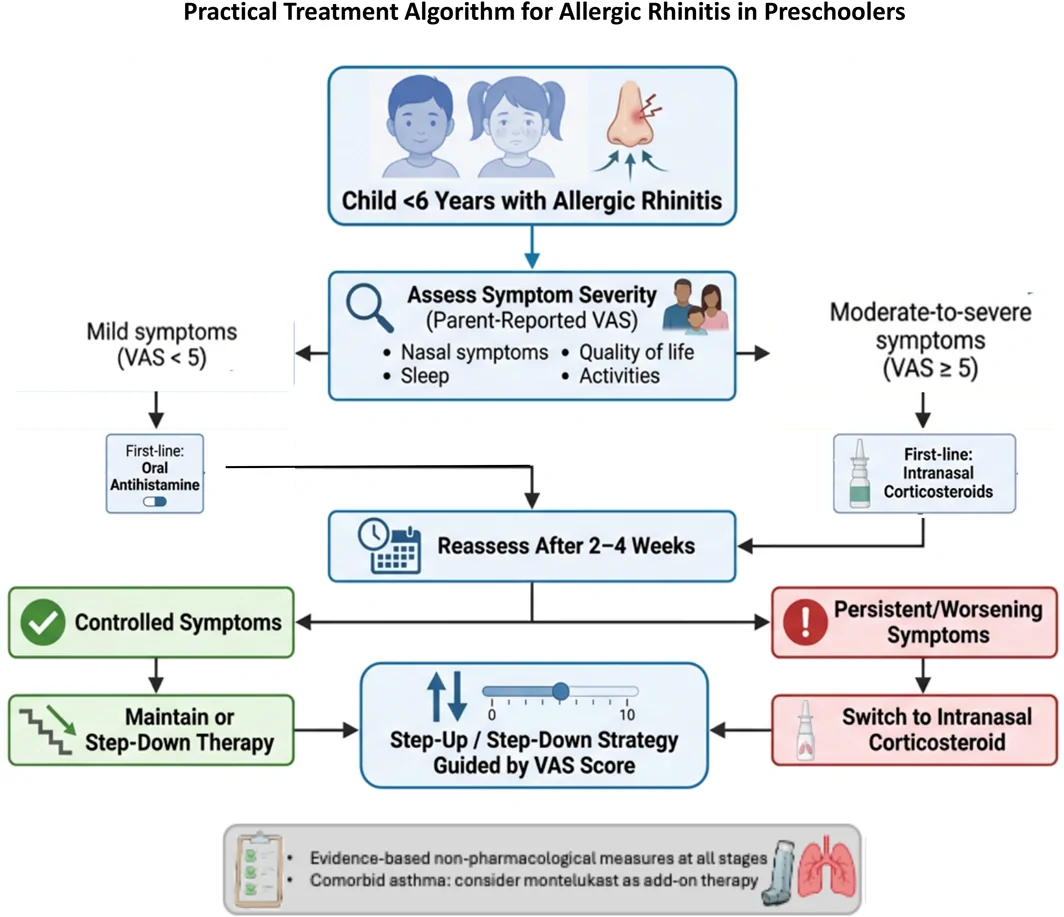
Practical algorithm in managing preschoolers.

### 12.2 Schoolchildren

Figure 7 shows a practical algorithm applied to children aged 6–11 years old. Symptom severity assessment is the first step in AR management. In this regard, schoolchildren can use VAS directly. Namely, VAS is a reliable patient‐reported outcome measure for assessing symptom perception in children [79]. Usually, as proposed by the ARIA guidelines, a VAS score of 10 indicates the worst symptom, whereas 0 indicates the absence of symptoms. However, the inverse scale is also reliable, and the orientation (vertical or horizontal) is irrelevant [79]. VAS assessment of nasal symptoms also correlates with the intensity of inflammation [80]. As a result, VAS assessment of AR has been validated by the ARIA and EUFOREA guidelines [81, 82]. In fact, these documents establish that a value of 5 or greater corresponds to moderate‐to‐severe symptoms and indicates poor control. A step‐up/step‐down approach is useful to manage treatment intensity appropriately. If symptoms are mild (VAS score < 5), the first‐line treatment is an oral antihistamine. After the initial reassessment (2–4 weeks), if symptoms persist, an intranasal corticosteroid may substitute for an oral antihistamine. In children with moderate‐to‐severe symptoms (VAS score ≥ 5), the combination of an oral antihistamine and an intranasal corticosteroid is a valuable option. Medication choice should be based on the age indication as reported in Tables 2 and 3. Non‐pharmacological remedies may be a valuable option. Allergen‐specific immunotherapy is a therapeutic option to consider. Asthma comorbidity may warrant additional montelukast.

**FIGURE 7:**
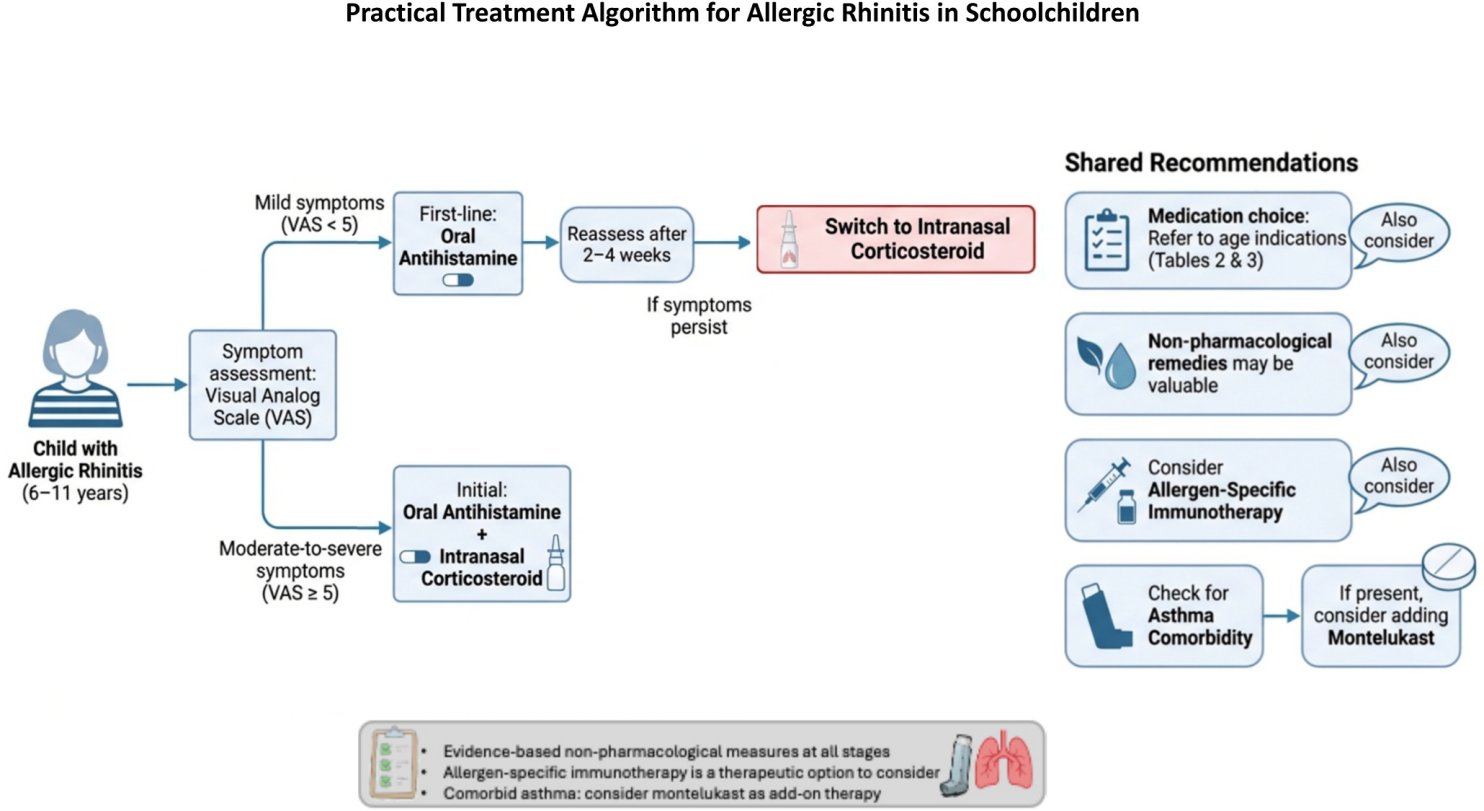
Practical algorithm in managing schoolchildren.

### 12.3 Adolescents

In adolescents, a VAS assessment is the first step in identifying the most appropriate treatment (Figure 8). Oral antihistamines are the first‐line treatment for mild AR. If symptoms persist at follow‐up, an intranasal corticosteroid may be preferred. In moderate‐to‐severe AR, the first option is an intranasal corticosteroid. If symptoms persist or worsen at follow‐up, a fixed‐combination intranasal antihistamine‐corticosteroid is the ideal choice. On the contrary, if symptom intensity diminishes, a step‐up may be observed. This approach may be followed for follow‐up. There are no age limitations in drug choice as reported in Tables 2 and 3.

**FIGURE 8:**
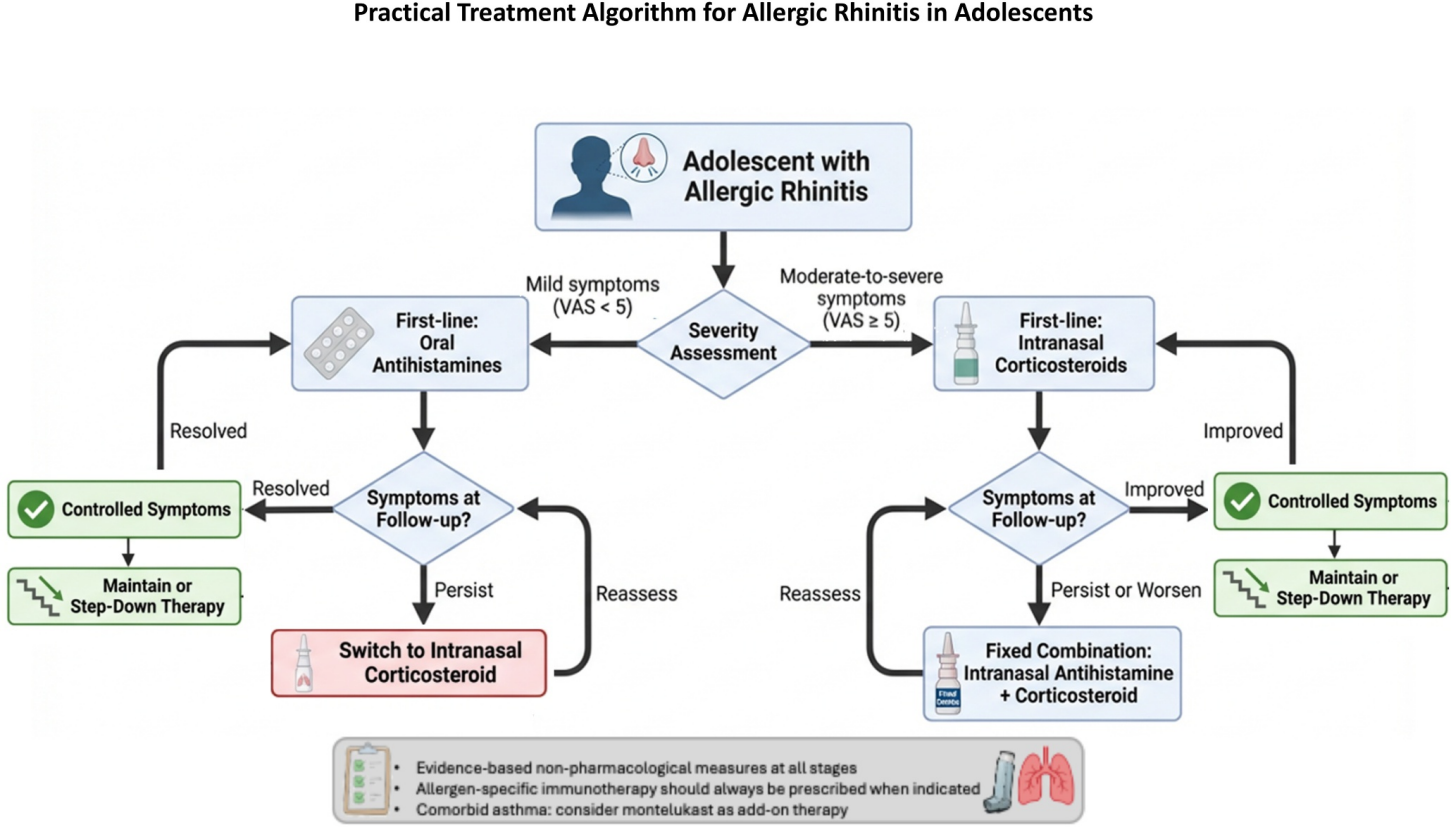
Practical algorithm in managing adolescents.

Regarding fixed‐combination antihistamine‐corticosteroid formulations, the azelastine‐fluticasone formulation has more clinical studies than the olopatadine‐mometasone formulation at present, as reported in a recent Point of View by SIAIP [39]. However, the combination of olopatadine and mometasone is also supported by robust RCTs [83, 84, 85]. Therefore, the efficacy and safety of both combinations have been documented by several RCTs. These combinations provide quick symptom relief and sustained anti‐inflammatory activity. Although both combinations are currently only indicated for adolescents, it is important to remember that there is also evidence for school‐age children (6–12 years old) [86, 87, 88, 89, 90]. Non‐pharmacological remedies may be considered as potential add‐on treatments. Finally, AIT should always be prescribed when indicated.

## 13 Conclusions and Future Perspectives

Allergic rhinitis represents one of the most prevalent chronic inflammatory conditions affecting the pediatric and adolescent population worldwide, with a substantial and often underestimated impact on quality of life, sleep architecture, academic performance, and comorbid conditions, including asthma and rhinosinusitis. Despite significant advances in understanding its pathophysiology and the ongoing refinement of therapeutic strategies, the practical management of AR in children and adolescents continues to pose unique clinical challenges that existing guidelines do not adequately address. Stepwise pharmacotherapy, primarily involving intranasal corticosteroids (INCS) and antihistamines (oral and/or intranasal), forms the cornerstone of treatment, with combination therapy (an intranasal antihistamine plus a corticosteroid) often indicated for moderate‐to‐severe cases [91]. Allergen immunotherapy (AIT), encompassing subcutaneous (SCIT) and sublingual (SLIT) forms, is a crucial disease‐modifying treatment. It offers long‐term symptom reduction, prevents new sensitizations, and may mitigate progression to asthma. Persistent research gaps include identifying novel biomarkers, developing specialized diagnostics for young children, and acquiring long‐term AIT efficacy data across diverse pediatric cohorts. This SIAIP document provides a series of practical recommendations and algorithms that may be adopted in clinical practice.

## Author Contributions

**Giorgio Ciprandi:** conceptualization, investigation, supervision, writing – original draft, writing – review and editing. **Amelia Licari:** data curation, writing – review and editing. **Cristiana Indolfi:** methodology, data curation. **Sara Manti:** data curation, writing – review and editing. **Michele Miraglia del Giudice:** methodology, supervision. **Maria Angela Tosca:** supervision, writing – review and editing. **Anna Maria Zicari:** methodology, data curation. **Gianluigi Marseglia:** conceptualization, supervision.

## Funding

The authors have nothing to report.

## Conflicts of Interest

The authors declare no conflicts of interest.

## Data Availability

The data that support the findings of this study are available from the corresponding author upon reasonable request.
